# Using reaction time and co-contraction to differentiate acquired (secondary) from functional ‘fixed’ dystonia

**DOI:** 10.1136/jnnp-2014-309040

**Published:** 2014-11-28

**Authors:** A Macerollo, A Batla, P Kassavetis, I Parees, K P Bhatia, M J Edwards

**Affiliations:** 1 Sobell Department of Motor Neuroscience and Movement Disorders, The National Hospital of Neurology and Neurosurgery, Institute of Neurology, University College London, London, UK; 2 Department of Basic Medical Sciences, Neuroscience and Sense Organs, Aldo Moro University of Bari, Bari, Italy

**Keywords:** EMG, DYSTONIA

Emphasis has been placed on the importance of making a positive diagnosis in functional (psychogenic) movement disorders. It has been suggested that ‘laboratory-supported criteria’ should be developed where electrophysiological and other tests can improve the level of certainty of diagnosis.^1^ Such criteria have been suggested for functional myoclonus^1^ and functional tremor.^2^ We report a preliminary study aimed at developing similar criteria for patients with ‘fixed’ dystonia (FD),^3^ a common presentation of functional dystonia.^4^

We performed surface electromyography (EMG) to assess the motor unit action potentials (MUAPs) in agonists and antagonists of the affected limb in patients with either functional FD (n=9, etable 1A), documented or clinically established following Fahn and Williams^4^ criteria, or acquired (n=9, etable 1B) dystonia (AD) due to brain lesions affecting an upper or lower limb. The inclusion criteria for the AD cases were the presence of brain lesions consistent with the clinical pattern of the dystonia.

We recorded EMG at rest and during a reaction time (RT) task where patients were asked to attempt to move in the opposite direction to the habitual limb posture after an auditory ‘go’ cue. Most participants had been receiving chronic treatment with botulinum toxin injections; however, they had not received the treatment for more than 3 months before the study. Dramatic immediate response to botulinum toxin is reported in similar patients. Participants were seated in a comfortable armchair. EMG was monitored with Ag/AgCl surface electrodes positioned on the agonist and antagonist muscles studied. The EMG activity was recorded at rest for 5 s, and then each trial included two auditory cues (100 ms, rise–fall time 20/20 ms, frequency 1000–5000 kHz). The initial sound was the warning stimulus and the second was the ‘go’ stimulus. The patients were asked to perform an isometric muscle contraction of muscles opposing the fixed posture as quickly as possible (contraction task) and to relax as soon as possible after the end of the go signal. The tasks involved flexion or extension of the wrist or plantar flexion or dorsiflexion depending on the muscles involved in the habitual posture. The go signal was delivered at five different intervals (2.5, 3, 3.5, 4 and 4.5 s) from the warning signal. The order of five states was pseudorandomised. The intertrial interval was greater than 15 s to avoid the effect of fatigue during the task and to allow for relaxation before each trial. For each patient, 70 trials were recorded. During recording, data were averaged and stored in a computer for off-line analysis. Data were analysed offline using Signal software (Cambridge Electronic Design, UK). The recordings were DC corrected and rectified. The RT and duration of MUAPs were measured manually for each trial. The amplitude of MUAPs was automatically computed. SPSS Statistics software (V.21.0.0) was used for the statistical analysis. Kolmogorov-Smirnoff test was used to measure the normality of the data distribution. When not normally distributed, the data were subjected to a log10 transformation. To compare the RT data between the two groups, we conducted an independent t test. Whereas to study the level of co-contraction in the two different diagnoses, we conducted a repeated measures multiway analysis of variance (ANOVA) on the data using the following factors: CONDITION (rest vs contractions), MUSCLE (agonist vs antagonist) and GROUP (FD vs AD). Post hoc tests were conducted with Bonferroni corrections for multiple comparisons. p Values less than 0.05 were considered to be significant.

Patients with AD had longer RT compared with patients with FD (238.9±50.3 vs 173.3±35.1 ms; t=−3.21, df=16; p=0.005; figure 1A). Although this measure separates the groups of patients, the scatter plot of the RTs for each patient indicates an overlap in the RT of the two groups (figure 1B). Repeated measures ANOVA (rmANOVA) of the ratio between EMG amplitude of agonist and the EMG amplitude of the antagonist with CONDITION (rest vs contraction) and MUSCLE (agonist vs antagonist) as within-subject factors and GROUP (FD vs SD) as between-subject factor showed an effect of MUSCLE (F_1,22.8_=1.11; p<0.001) and CONDITION (F_1,82.42_=12.07; p<0.001). In addition, there was a significant MUSCLE×CONDITION interaction (F_1,38.53_=1.67; p<0.001) and MUSCLE×DIAGNOSIS interaction (F_1,18.74_=0.91; p=0.001). There was no significant effect of diagnosis (F_1,0.56_=0.25; p>0.05). Post hoc tests with Bonferroni correction showed no differences in EMG activity between FD and AD groups at rest.

**Figure 1:**
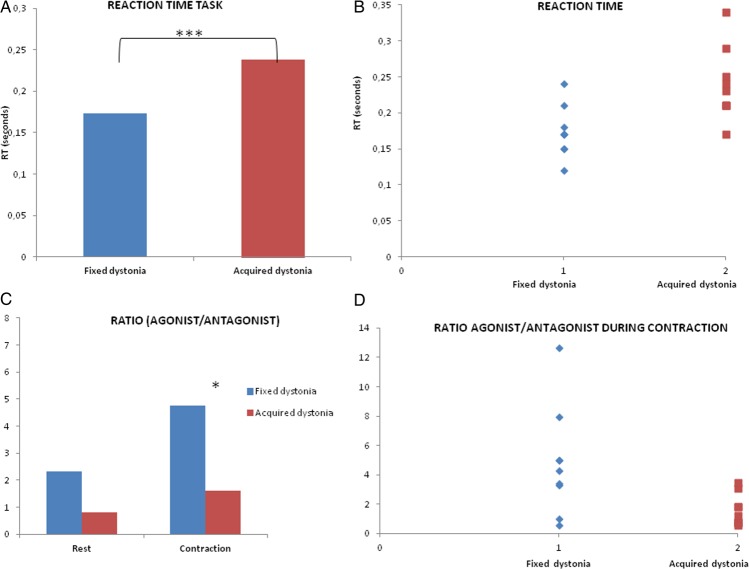
Mean values of the reaction times (RT) during the contraction tasks in the agonist muscles of patients with fixed dystonia and acquired dystonia. Statistical significance (unpaired t tests): ***p<0.01 (A). Scatter plots of RT for each patient (B). Ratio amplitude of electromyography (EMG) activity between agonist and antagonist muscles involving in the task at rest and during voluntary movement opposing to fixed posture, statistical significance: *p<0.05 (C). Scatter plots of EMG amplitude agonist/antagonist ratio during contraction for each patient (D).

To explore the difference in the level of co-contraction in the two groups, we performed a post hoc rmANOVA of EMG amplitude ratio between agonist and antagonist during the two conditions (at rest and during contraction). There was a significant effect of the CONDITION (F_1,14.90_=0.65; p=0.002) and a significant effect of the DIAGNOSIS (F_1,4.8_=0.92; p=0.04). Post hoc analysis showed that the EMG amplitude was higher during contraction in both groups and the ratio was higher in patients with FD than in those with AD; that is, patients with FD have *less* co-contraction than patients with AD (figure 1C). Despite this group difference, the scatter plot of the ratio between agonist/antagonist EMG activities for each patient showed overlap in the values of the two groups (figure 1D).

Reliable clinical diagnosis of functional FD is known to be challenging. We report an EMG study to explore RT and co-contraction as possible measures in this regard in functional dystonia compared with a form of organic dystonia that causes fixed postures.

We found a long RT in patients with AD, similar to previous results in primary dystonia.^5^ Patients with FD showed RT values similar to those reported in historical cohorts of healthy patients.^5^ Furthermore, patients with FD had lower levels of co-contraction during a voluntary movement compared to those with AD. Notably, the individual data on RT and co-contraction show clear overlap between the two groups. Thus, even if these two parameters can be useful to differentiate at a group level, they are not suitable in this form as diagnostic criteria. Moreover, we did not find a pattern of response that was abnormal in a different manner to AD (eg, failure to activate the muscles requested), which would have allowed a more positive differentiation of FD from AD.

Although the findings of our study show promise in better classifying this common disorder, we appreciate the limitation of lack of a healthy control group in the investigation. These data therefore represent an initial attempt to provide laboratory criteria for FD, but there is clearly still an unmet need for a positive laboratory test to support clinical diagnosis in FD.

## Supplementary Material

Web supplement
