# Supplementary material for: Using reaction time and co-contraction to differentiate acquired (secondary) from functional ‘fixed’ dystonia
Source: J Neurol Neurosurg Psychiatry. 2014 Nov 28;86(8):933–4. doi: 10.1136/jnnp-2014-309040 (PMC4516003; doi:10.1136/jnnp-2014-309040)
Supplement: Web supplement [file jnnp-2014-309040-s1.pdf]

e-Table 1A Clinical and demographic characteristics of patients with fixed dystonia

Onset age	Disease Duration (years)	Mode of onset	Preceding event	Findings	The Burke-Fahn-Marsden rating score for dystonia-Movement section*	Comorbidities	Response to botulinum toxin
30	11	Acute	Pelvic surgery	Both feet in turned and plantar flexed	18	Joint hypermobility, urinary dysfunction, menstrual irregularity, anxiety	Immediate with partial remission
24	6	Acute	Hip surgery and fall during recovery	Right hip internal rotation with plantar flexed and inversion at foot	8	Joint hypermobility, urinary dysfunction, menstrual irregularity, anxiety	Immediate with partial remission
32	8	Acute	Right Foot injury	Right foot inversion and plantar flexion	7	Hypermobility, urinary dysfunction, depression	Immediate with partial remission
36	10	Acute	Long standing right sesmoid bone fracture, immobilized	Right foot inversion and plantar flexion	8	Panic disorder	Immediate with partial remission
45	1	Acute	Left foot injury	Left foot inversion	7	Urinary dysfunction	Immediate with partial remission
38	4	Acute	Left foot trauma	Left foot plantar flexion and slight eversion	7	None	Not injected till the time of evaluation
46	1	Acute	Right foot injury	Right foot plantar flexion and inversion	9	None	Not injected till the time of evaluation
35	14	Acute	Pelvic surgery	Fixed posture of right hand and right leg	8	Migraine, fibromyalgia and restless leg syndrome	Immediate with partial remission
38	6	Acute	Right foot injury	Right foot plantar flexion and inversion	9	None	Immediate with partial remission

(*Reference Burke RE et al. Neurology. 1985;35:73-77)

e-Table 1B Clinical and demographic characteristics of patients with acquired dystonia

Onset age (years)	Disease Duration (years)	Mode of onset	Preceding event	Findings	The Burke-Fahn-Marsden rating score for dystonia-Movement section*	Comorbidities	Response to botulinum toxin
15	9	Progressive	Stroke (lesion in the right lentiform nucleus)	Left hand dystonia	8	None	Poor
Birth	66	Progressive	Cerebral Paralysis	Left hemi dystonia	16	None	No response
14	4	Progressive	Stroke (lesion in the left lentiform nucleus)	Right foot dystonia	7	None	Poor
54	4	Progressive	Significant cervical canal stenosis with 2 level decompression	Dystonic posturing affecting the right arm, particularly the hand. Some abnormal posturing present in the right leg.	8	None	Poor
Birth	57	Progressive	Cerebral Paralysis	Right hemidystonia	12	None	Poor
1	43	Progressive	Encephalitic illness	Right hemidystonia	12	None	Poor
39	27	Progressive	lumbar surgery to “remove a spur”	Left foot dystonia	7	None	Poor
Birth	22	Progressive	Left cerebellar hypoplasia	Left hemidystonia	12	None	Poor
Birth	21	Progressive	Cerebral Paralysis	Left hemidystonia	14	None	Good

(*Reference Burke RE et al. Neurology. 1985;35:73-77)
